# Alterations in the Expression of the NF-κB Family Member RelB as a Novel Marker of Cardiovascular Outcomes during Acute Exacerbations of Chronic Obstructive Pulmonary Disease

**DOI:** 10.1371/journal.pone.0112965

**Published:** 2014-11-19

**Authors:** Laura Labonté, Patrick Coulombe, Michela Zago, Jean Bourbeau, Carolyn J. Baglole

**Affiliations:** 1 Department of Medicine, McGill University, Montreal, Quebec, Canada; 2 Respiratory Epidemiology and Clinical Research Unit (RECRU), Montreal Chest Institute, McGill University Health Centre, Montreal, Quebec, Canada; 3 Meakins-Christie Laboratories, McGill University, Montreal, Quebec, Canada; University of Rochester Medical Center, United States of America

## Abstract

**Background:**

Chronic obstructive pulmonary disease (COPD) exacerbations are acute events of worsened respiratory symptoms and enhanced inflammation partly mediated by NF-κB activation. RelB, an NF-κB family member, suppresses cigarette smoke-induced inflammation but its expression in COPD is unknown. Moreover, there is no information on its association with clinical features of COPD. The objectives of this study were to assess RelB expression relative to markers of inflammation as well as its association with cardiovascular and pulmonary features of COPD patients at stable-state and exacerbation.

**Methods:**

Data from 48 COPD patients were analyzed. Blood samples were collected from stable-state and exacerbating patients. After RNA isolation, quantitative real-time polymerase chain reaction (qRT-PCR) was performed to assess RelB, Cox-2, IL-8 and IL-1β mRNA expression and their associations with measured clinical variables.

**Results:**

Of the 48 COPD subjects, 18 were in stable-state and 30 were in exacerbation. RelB mRNA expression was lower than that of Cox-2, IL-8, and IL-1β in all cases (all p<0.001, except for IL-8 at exacerbation (p = 0.22)). Cox-2, IL-8 and IL-1β were significantly associated with clinical features of patients in both stable-state and at exacerbation. There was no association with RelB expression and any clinical features in COPD subjects at stable-state. RelB mRNA levels were significantly associated with cardiovascular events such as systolic blood pressure during exacerbation.

**Conclusions:**

RelB mRNA expression is lower than that of the other inflammatory mediators. Expression of Cox-2, IL-8 and IL-1β were related to clinical features in both stable-state and at exacerbation. However, RelB expression was associated with clinical features of patients only during exacerbation, suggesting that RelB may represent a novel marker of health outcomes, in particular cardiovascular, during exacerbation in COPD.

## Introduction

Chronic obstructive pulmonary disease (COPD) is characterized by progressive, not fully reversible airflow limitation [1] and chronic inflammation [2]. The course of COPD is perturbed by acute events of worsened respiratory symptoms known as exacerbations [3]–[7]. Exacerbations are associated with heightened pulmonary and systemic inflammatory responses [8], and lead to significant morbidity and mortality, especially due to cardiovascular events, as well as decreased health-related quality of life and an accelerated decline in lung function [3], [4], [9]–[11].

COPD inflammation in stable-state and at exacerbation is partly mediated by nuclear factor-κB (NF-κB) activation [12]-[14]. NF-κB is a ubiquitous transcription factor family composed of five proteins [15] activated by cigarette smoke (CS) that collectively are involved in the regulation of gene expression for pro-inflammatory cytokines, chemokines and adhesion molecules [12], [16]. These include interleukin (IL)-1β, a key orchestrator of the immune response in COPD [14] that can activate NF-κB and is produced by airway epithelial cells in response to CS or acute injury [14], [17]. Other mediators include IL-8, which is involved in inflammatory cell recruitment in COPD [18]–[21], especially with bacterial infection at exacerbation [20], [21]; and cyclooxygenase-2 (COX-2), an inducible enzyme that catalyzes arachidonic acid transformation into thromboxane and prostaglandins [12]. COX-2 induction can also occur via IL-1β activation of the NF-κB pathway and is enhanced in response to noxious stimuli such as CS [12], [22].

Although activation of NF-κB is typically regarded as pro-inflammatory, we recently identified RelB, another member of the NF-κB family, as a potent suppressor of CS-induced inflammation [23]–[27]. Weih *et al*. [28], [29] showed that mice deficient in RelB have severely increased multi-organ inflammation, suggesting that RelB may have a role in suppressing inflammation. Our *in-vitro* and *in-vivo* data provide further evidence for an anti-inflammatory role for RelB. We have shown that loss of RelB expression due to smoke exposure promotes pro-inflammatory mediator production (including IL-8 and COX-2 expression), whereas RelB reconstitution reduces inflammation associated with CS [23], [24]. Reciprocally, overexpression of pulmonary RelB in mice exposed to CS is associated with decreased lung neutrophil infiltration, pro-inflammatory cytokine and chemokine production and COX-2/prostaglandin production [25].

Despite increasing experimental evidence regarding the anti-inflammatory abilities of RelB against CS, neither expression of RelB nor its expression relative to other inflammatory mediators has been studied in the context of COPD at stable state or during exacerbation.

It is also unknown whether RelB is associated with any clinically-relevant outcomes in COPD, including acid-base balance and cardiovascular events. Recent experimental evidence in human lung epithelial cells has shown that increased nuclear RelB correlates with hypercapnia-induced protection against lung injury. This suggests that RelB is carbon dioxide-sensitive and may contribute to the benefits of hypercapnia in pulmonary inflammatory diseases [30]. Given that in COPD acid-base disturbances are common, particularly during exacerbations [31] and are associated with poor health outcomes, we hypothesize that RelB may be associated with clinical variables involved in acid-base maintenance during COPD and its exacerbations. It is also well-described that the period following COPD exacerbations has been associated with enhanced risk of acute cardiovascular events [32]–[34] possibly due to increased inflammation. Experimental evidence supports a protective role for RelB in the cardiovascular system, including our data where RelB controls pulmonary endothelial intercellular adhesion molecule-1 (ICAM-1) levels in response to CS [27]. RelB-deficient mice also have inflammatory cell infiltrates in the heart [35]. Thus our data and that of others support that RelB expression is an essential suppressor of pulmonary and cardiovascular inflammation. We therefore hypothesize that systemic RelB expression will be associated with cardiovascular outcomes in COPD, particularly at exacerbation when inflammation is typically increased.

The objectives of this study were: (1) to assess systemic RelB mRNA expression relative to other inflammatory markers relevant to both COPD pathogenesis and whose expression is regulated by RelB (*e.g.* Cox-2, IL-8, IL-1β) in stable-state and exacerbating COPD patients and (2) to assess associations between these two subject groups in relation to acid-base, cardiovascular and pulmonary patient variables. Our results reveal for the first time that RelB is expressed in stable-state and at exacerbation in COPD. Although circulating RelB levels did not correlate with any clinical parameters at stable-state, we show for the first time that RelB expression is associated with both acid-base and cardiovascular features of COPD patients at exacerbation, suggesting that RelB may represent a novel biomarker of cardiovascular outcomes during COPD exacerbations.

## Materials and Methods

### Study subjects

48 COPD patients were recruited at the Montreal Chest Institute between February-September 2013, including 18 in stable-state (no-exacerbation in the 4 weeks before assessment) and 30 who were hospitalized with a primary diagnosis of exacerbation. Exacerbations were defined based on symptom-change lasting at least two consecutive days requiring treatment with corticosteroid and/or antibiotics and hospitalization [36], [37]. All subjects had to be ≥40 years old, previously diagnosed with COPD [1] (post-bronchodilator forced expired volume in one second (FEV_1_) to forced vital capacity (FVC) ratio <0.70) and have clinical evidence of cardiovascular disease and/or established risk factors (clinical signs or imaging studies; coronary artery disease; peripheral vascular disease; previous stroke or myocardial infarction (MI); diabetes mellitus with target organ disease; or treatment for hypercholesterolemia, hypertension, diabetes mellitus or peripheral vascular disease). This study was conducted in accordance with the amended Declaration of Helsinki. The McGill University Faculty of Medicine Institutional Review Board (IRB) approved the protocol and written informed consent was obtained from all patients.

### Clinical assessment and blood sample collection

Subjects underwent post-bronchodilator spirometry, blood collection (biomarker, lipid profile, complete cell blood count, and metabolic panel analyses), pulse, oxygen saturation, capillary gas, and hemodynamic measurements (carotid-femoral pulse wave velocity (cfPWV) and pulse wave analysis). Peripheral blood was collected using PAXgene blood RNA tubes (PreAnalytiX GmbH, Hombrechtikon, Switzerland). For those in stable-state, assessments occurred during respiratory clinic visits and for exacerbating subjects, assessments were made during the first 72 hours of hospitalization. Samples were frozen at −80 until analysis.

### Analysis of gene expression

RNA was isolated using PAXgene blood RNA kits (PreAnalytiX GmbH), and quantified using a Nanodrop 1000 spectrophotometer (Thermo Fisher Scientific, Wilmington, Delaware). Reverse transcription of total RNA was carried out using iScriptII Reverse Transcription Supermix (Bio-Rad Laboratories, Mississauga, Canada). Quantitative real-time polymerase chain reaction (qRT-PCR) was performed by adding 1 µL cDNA and 0.5 mM primers (Table 1) to SsoFast EvaGreen (Bio-Rad). PCR amplification was performed using a CFX96 Real-Time PCR Detection System (Bio-Rad). Melt curves were analyzed to confirm that non-specific products were absent. The fluorescence detection threshold was set above the non-template control background within the linear phases of PCR amplifications and the cycle threshold (Ct) of each reaction was detected. Gene expression was analyzed using the ΔΔCt method and results are presented as fold-differences normalized to housekeeping gene (β-actin).

**Table 1:** qRT-PCR Primer sequences.

Primer	Forward sequence	Reverse sequence
hCox-2	TCACAGGCTTCCATTGACCAG	CCGAGGCTTTTCTACCAGA
hIL-8	GATGTCAGTGCATAAAGACATACTCCAAAC	GCTCTCTTCCATCAGAAAGCTTTACAATAA
hIL-1β	AAAAGCTTGGTGATGTCTGGTCCATATGAA	CTTATCATCTTTCAACACGCAGGACAGGTA
hRelB	TGTGGTGAGGATCTGCTTCCA G	GGCCCGCTTTCCTTGTTAATT C
β-actin	CTACAATGAGCTGCGTGTG	TGGGGTGTTGAAGGTCTC

### Statistical analysis

Analyses were performed using SAS version 9.3 software (SAS Institute. Inc., Cary, North Carolina). For mean comparisons, two-tailed T-tests (normal distribution) or Wilcoxon signed-rank tests (non-normal distribution) were used; Chi-squared tests were used for dichotomous variables. A p-value of 0.05 or less was deemed statistically significant. Associations between clinical variables and mediators were assessed using Pearson correlation coefficients. Multiple regression models were used to determine whether changes in mediator expression could predict changes in clinical variables (adjusted for age, sex, body mass index and smoking history).

## Results

### Patient characteristics

Both the stable-state and exacerbation subjects groups were statistically-similar in most of the clinical features examined except that the hospitalized group had a lower mean FEV_1_ (% predicted and liters), included more current smokers, and had significantly greater blood pressure and elevated heart rate (Table 2).

**Table 2:** Patient characteristics.

	Stable-state (n = 18)	Exacerbation (n = 30)	P-value
Mean age (years)	71.0	71.1	0.86
No. Male (%)	7 (39)	14 (47)	0.11
Mean FEV_1_ % predicted	43.5	34.5	0.046*
Mean FEV_1_ L	1.1	0.79	0.0082*
Mean FEV_1_/FVC	0.44	0.48	0.30
Smoking status, n(%)			
Ex-smoker	17 (94)	23 (77)	0.083
Current smoker	1 (6)	7 (23)	0.014*
LTOT (%)	22	20	0.62
Mean pack-year smoking history	68.77	57.84	0.42
Mean body mass index	27.9	25.1	0.15
Mean no. reported exacerbations in past 12 months	2	3	0.57
Mean systolic BP	111	125	0.013*
Mean diastolic BP	54	66	0.0049*
Mean heart rate	78	92	0.0030*
Pulse oximetry (%)	94	94	0.57

*Denotes statistical significance. No: number, LTOT: long-term oxygen therapy, BP: blood pressure.

### Stable-state and exacerbation inflammatory mediator expression relative to RelB levels

**Figure 1:**
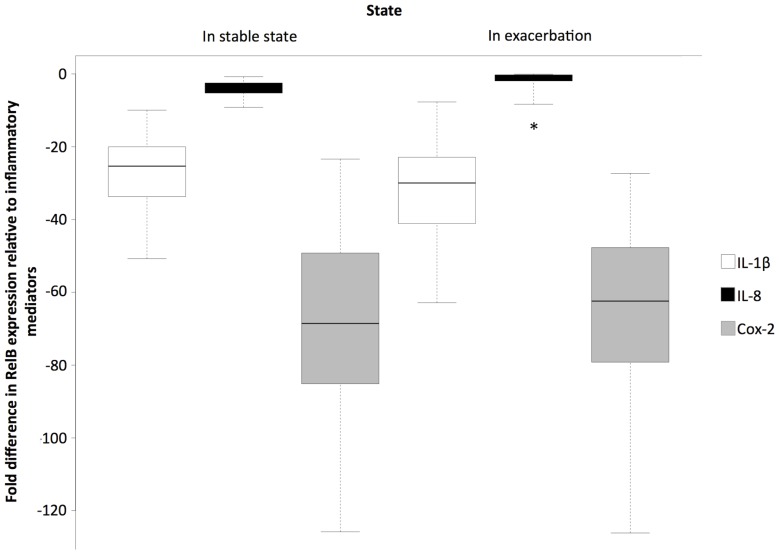
Fold difference in RelB mRNA expression relative to IL-1β, IL-8 and Cox-2 mRNA expression for patients in stable-state (n = 18) and those in exacerbation (n = 30). * Denotes non-statistically significant difference in expression (p = 0.22), for all other p<0.001.

Figure 1 shows the relative fold-difference in mRNA expression of RelB compared to Cox-2, IL-8 and IL-1β. When considering the Stable-state group only, RelB mRNA expression was 69.01 fold less than that of Cox-2, 4.42 fold less than that of IL-8 and 26.97 fold less than that of IL-1β (p<0.001 for all cases). When considering the subjects who were in exacerbation, RelB mRNA expression was 65.46 fold less than that of Cox-2 (p<0.001), 1.48 fold less than that of IL-8 (p = 0.22) and 32.16 fold less than that of IL-1β (p<0.001). Moreover in exacerbating patients, RelB mRNA expression was 1.43 fold lower than in stable-state patients (p<0.001) (Figure S1).

### Associations between inflammatory mediators and patient clinical features during Stable-state or Exacerbation

In the stable-state patients (Table 3), Cox-2 mRNA expression correlated negatively with FEV_1_ (liters), FEV_1_/FVC and the ratio of cholesterol to high-density lipoprotein (HDL). IL-8 mRNA correlated negatively with systolic and diastolic blood pressure, and absolute basophil count, and positively with red blood cell (RBC) diameter width. IL-1β mRNA expression correlated positively with pack-year smoking history, white blood cell (WBC) count, absolute monocyte count and absolute neutrophil count. RelB mRNA expression was not correlated to any clinical variables in stable-state.

**Table 3:** Associations between inflammatory mediators and clinical features of patients in stable-state and at exacerbation.

	Inflammatory mediators							
	Cox-2 expression levels		IL-1β expression levels		RelB expression levels		IL-8 expression levels	
	Correlation coefficients		Correlation coefficients		Correlation coefficients		Correlation coefficients	
Clinical features	Stable-state n = 18	Exacerbation n = 30	Stable-state n = 18	Exacerbation n = 30	Stable-state n = 18	Exacerbation n = 30	Stable-state n = 18	Exacerbation n = 30
Pack-year smoking history	0.36	0.39*	0.49*	0.13	0.39	−0.020	0.050	0.18
Systolic BP	−0.18	−0.42*	0.13	−0.32	−0.090	−0.41*	−0.52*	−0.52*
Diastolic BP	−0.37	−0.29	−0.060	−0.36	−0.26	−0.17	−0.51*	−0.10
FEV_1_ (L)	−0.51*	−0.060	0.11	0.070	−0.19	−0.080	−0.25	−0.20
FEV_1_/FVC	−0.56*	−0.11	−0.33	−0.10	−0.37	−0.12	−0.11	−0.17
pH	0.33	0.11	0.42	0.22	0.16	−0.21	0.58	−0.46*
PCO_2_ (mmHg)	−0.21	0.26	−0.34	−0.090	-0.10	0.41	−0.13	0.49*
Ca++ (mmol/L)	−0.070	0.47*	0.15	0.40	−0.57	0.35	0.080	0.19
Cholesterol/HDL	−0.51*	−0.060	−0.30	−0.15	0.080	0	−0.090	0.070
Cholesterol (mmol/L)	−0.25	−0.11	−0.34	−0.41*	−0.050	−0.36	−0.25	0.050
Aix (%)	−0.10	−0.15	−0.29	−0.13	−0.11	0.22	−0.17	0.46*
Hct (%)	0.26	−0.47*	0.48	−0.38	−0.030	−0.13	−0.10	−0.30
Hct (L/L)	−0.21	−0.36	0.30	−0.52*	−0.24	−0.11	−0.44	−0.18
WBC^a^	0.24	0.010	0.76**	0.33	0.32	−0.040	0.40	−0.12
RBC (10∧12/L)	−0.15	−0.33	0.31	−0.47*	−0.11	−0.14	−0.37	−0.18
RBC diameter width (cV)	0.030	0.52*	0.19	0.29	0.26	0.22	0.67*	0.050
Hemoglobin (g/L)	−0.14	−0.34	0.30	−0.46*	−0.21	−0.16	−0.45	−0.17
Abs. MNC^a^	0.070	−0.05	0.63*	0.080	0.31	0.090	0.28	−0.060
Abs. neutrophil^a^	0.32	0	0.74**	0.35	0.29	−0.10	0.46	−0.15
Abs. basophil^a^	−0.020	0.25	0.21	0.20	−0.060	0.32	−0.61*	0.080
Abs. eosinophil^a^	0.010	0.49*	0.010	0.050	0.20	0.30	0.20	0.55*
Anion gap (mmol/L)	−0.050	−0.47*	0.13	0.020	0.21	−0.55*	0.15	−0.55*
Glucose (mmol/L)	−0.33	−0.31	0.14	0.15	0.050	−0.41*	0.12	−0.39*

Pearson correlation coefficients were used to determine associations.

*Denotes statistical significance p<0.05, ** denotes statistical significance p<0.001.

a units are (10∧9/L).

BP: blood pressure, Ca++: calcium, Hct: hematocrit, Abs.: absolute, MNC: monocyte.

When considering mRNA expression in exacerbating subjects (Table 3), Cox-2 mRNA expression correlated negatively with systolic blood pressure, hematocrit (%) and anion gap, and positively with pack-year smoking history, calcium levels, RBC diameter width and absolute eosinophil count. IL-8 mRNA expression correlated negatively with systolic blood pressure, pH, anion gap and glucose level, and positively with the partial pressure of carbon dioxide (PCO_2_), augmentation index (AIx) and absolute eosinophil count. IL-1β mRNA expression correlated negatively with cholesterol level, RBC count, hemoglobin level and hematocrit level. RelB mRNA expression correlated negatively with systolic blood pressure, anion gap, and glucose level.

### Predictors of change in patient clinical features

**Figure 2:**
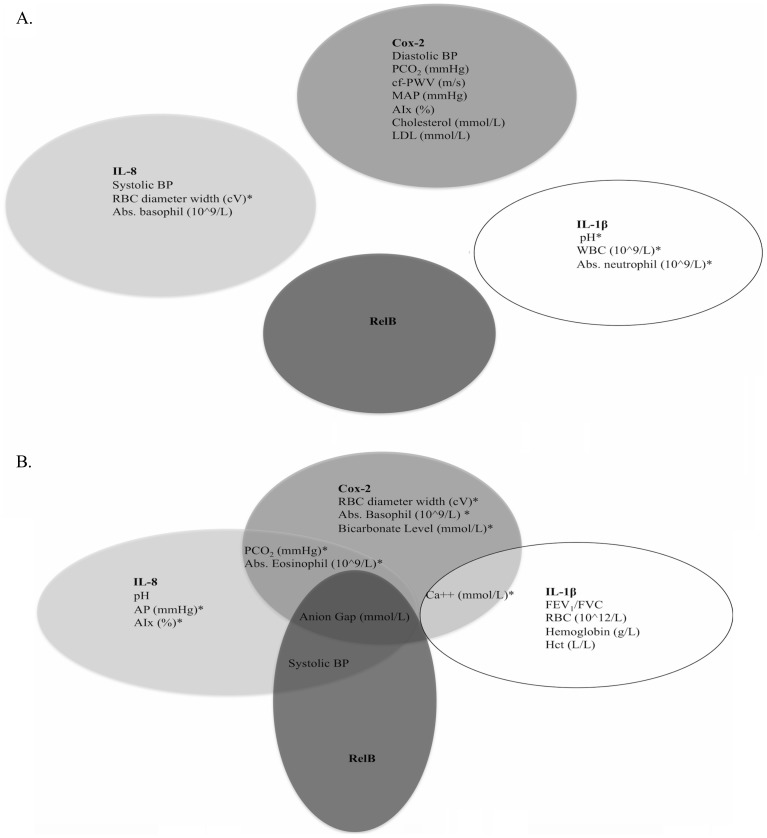
Ability of inflammatory mediators to predict changes in patient clinical features in a) stable-state (n = 18) and b) at exacerbation (n = 30). A linear regression model was used and adjusted for age, sex, body mass index and smoking pack-years. The inflammatory mediators were used as independent variables to predict changes in all assessed clinical features. * Denotes a positive association. BP: blood pressure, MAP: mean arterial pressure, Abs: absolute, AP: augmentation pressure.

When only considering the predictive relationships between inflammatory mediator mRNA expression and clinical features of stable-state patients (Figure 2a; Table 4), Cox-2 could predict negative changes in diastolic blood pressure, PCO_2_, cfPWV, mean arterial pressure, AIx, cholesterol level, and low density lipoprotein (LDL). IL-8 expression could predict negative changes in systolic blood pressure and absolute basophil count, and positive changes in RBC diameter width. IL-1β expression could predict positive changes in pH, WBC count, and absolute neutrophil count. The expression of RelB could not significantly predict changes for any variables in stable-state.

**Table 4:** Predictors of clinical feature change in stable-state.

	Stable-state COPD (n = 18)							
	Cox-2 expression levels		IL-1beta expression levels		RelB expression levels		IL-8 expression levels	
Clinical features	ß (95% CI)	p-value	ß (95% CI)	p-value	ß (95% CI)	p-value	ß (95% CI)	p-value
Systolic BP	−637.13 (−1839.06–564.79)	0.27	48.89 (−2856.79–2954.56)	0.97	−66325.7 (−193597–60945.40)	0.28	−8410.48 (−16147.2–−673.76)	0.036*
Diastolic BP	−1173.04 (−2117.53–−228.56)	0.019*	−391.22 (−3170.54–2388.10)	0.76	−70756.5 (−191298–49784.85)	0.22	−7471.46 (−15178.5–235.53)	0.056
pH	2.85 (−0.64–6.33)	0.086	8.24 (1.35–15.13)	0.029*	182.37 (−357.62–722.36)	0.40	28.54 (−6.07–63.15)	0.084
PCO2 (mmHg)	−474.44 (−861.41–−87.47)	0.027*	−593.12 (−2343.89–1157.64)	0.40	−15073.0 (−98917.2–68771.15)	0.64	−1125.91 (−8586.88–6335.06)	0.70
cfPWV (m/s)	−634.31 (−1006.47–−262.14)	0.0030*	−807.59 (−1798.15–182.97)	0.10	−10424.8 (−62823.5–41973.93)	0.67	−557.57 (−4426.40–3311.26)	0.76
MAP (mmHg)	−1445.68 (−2228.01–−663.36)	0.0020*	−616.78 (−3197.05–1963.49)	0.60	−61331.8 (−172971–50307.08)	0.25	−5757.05 (−13787.5–2273.42)	0.14
Aix %	−931.72 (−1859.35–−4.09)	0.049*	−546.46 (−2782.59–1689.67)	0.59	−37079.5 (−138077–63918.39)	0.43	−1724.41 (−9533.71–6084.89)	0.63
Cholesterol (mmol/L)	−62.21 (−119.63–−4.80)	0.036*	−65.24 (−282.03–151.55)	0.52	86.86 (−8457.91–8631.63)	0.98	−302.84 (−1043.82–438.15)	0.38
LDL (mmol/L)	−61.38 (−117.57–−5.19)	0.035*	−85.14 (−294.24–123.95)	0.39	1469.95 (−6853.37–9793.26)	0.70	−319.33 (−1042.00–403.34)	0.35
WBC (10∧9/L)	85.65 (−54.29–225.59)	0.21	504.49 (186.88–822.10)	0.0050*	3843.09 (−12921.6–20607.79)	0.62	913.43 (−368.64–2195.49)	0.15
RBC diameter width (cV)	44.43 (−136.38–225.24)	0.60	270.73 (−259.38–800.83)	0.29	8895.13 (−10807.2–28597.48)	0.34	1690.40 (386.50–2994.31)	0.016*
Abs. neutrophil (10∧9/L)	85.84 (−51.73–223.41)	0.20	470.02 (138.86–801.18)	0.010*	2751.54 (−13865.3–19368.40)	0.72	1021.97 (−200.52–2244.47)	0.093
Abs. basophil (10∧9/L)	−1.23 (−4.08–1.62)	0.36	−2.48 (−11.38–6.43)	0.55	−111.46 (−435.83–212.92)	0.47	−27.49 (−48.50–−6.48)	0.015*

Linear regression models were used to estimate predictors of change in clinical features and were adjusted for age, sex, body mass index, and smoking pack-years. Inflammatory markers were used as independent variables to predict changes in all clinical features of patients. ß represents the change in a clinical feature associated with a one-unit change in biomarker expression.

*Denotes statistical significance.

MAP: mean arterial pressure.

For exacerbating patients (Figure 2b; Table 5), Cox-2 mRNA expression could predict negative changes in anion gap and positive changes in PCO_2_, blood calcium level, RBC width, absolute eosinophil count, absolute basophil count and bicarbonate level. IL-8 mRNA expression could predict negative changes in systolic blood pressure, pH, and anion gap, and positive changes in PCO_2_, augmentation pressure, AIx and absolute eosinophil count. IL-1β expression could predict negative changes in FEV_1_/FVC, RBC count, hemoglobin level, and hematocrit level, and positive changes in calcium level. RelB mRNA expression could predict negative changes in systolic blood pressure and anion gap.

**Table 5:** Predictors of clinical feature change at exacerbation.

	Exacerbation (n = 30)							
	Cox-2 expression levels		IL-1β expression levels		RelB expression levels		IL-8 expression levels	
Clinical features	ß (95% CI)	p-value	ß (95% CI)	p-value	ß (95% CI)	p-value	ß (95% CI)	p-value
Systolic BP	−677.58 (−1586.61–231.45)	0.14	−1390.54 (−3283.70–502.62)	0.14	−50749.6 (−92792.3–−8706.83)*	0.020*	−12649.1 (−24031.7–−1266.55)*	0.031*
pH	−0.13 (−2.15–1.90)	0.90	1.00 (−2.94–4.94)	0.60	−25.42 (−116.18–65.33)	0.56	−29.07 (−52.47–−5.67)*	0.018*
PCO_2_ (mmHg)	360.06 (18.51–701.60)	0.040*	123.24 (−620.68–867.17)	0.73	12518.77 (−3831.36–28868.91)	0.13	5085.44 (564.18–9606.71)*	0.030*
Ca++ (mmol/L)	2.82 (0.46–5.19)	0.023*	5.41 (0.34–10.48)*	0.038*	85.87 (−33.15–204.90)	0.14	15.66 (−23.12–54.43)	0.40
FEV_1_/FVC	−2.27 (−9.08–4.55)	0.50	−14.75 (−28.27–−1.23)*	0.034*	−157.61 (−492.65–177.42)	0.34	−46.09 (−140.18–48.00)	0.32
AP (mmHg)	−115.51 (−500.67–269.66)	0.54	−120.55 (−831.09–589.99)	0.73	12466.04 (−5030.98–29963.05)	0.15	5254.52 (1644.29–8864.76)*	0.0070*
Aix (%)	−348.85 (−1173.38–475.68)	0.39	−572.74 (−2098.13–952.65)	0.44	22125.77 (−16420.3–60671.88)	0.24	11956.20 (4295.85–19616.55)*	0.0040*
RBC (10∧12/L)	−26.85 (−54.28–0.57)	0.060	−79.50 (−132.82–−26.18)*	0.0060*	−833.40 (−2267.21–600.42)	0.24	−233.26 (−634.42–167.91)	0.24
Hemoglobin (g/L)	−697.08 (−1584.64–190.48)	0.12	−2074.77 (−3864.48–−285.06)*	0.025*	−27604.0 (−72519.4–17311.45)	0.22	−5718.07 (−18463.6–7027.43)	0.36
Hct (L/L)	−2.18 (−4.87–0.51)	0.11	−7.33 (−12.46–−2.19)*	0.0070*	−63.61 (−202.16–74.94)	0.35	−17.94 (−56.53–20.65)	0.34
RBC diameter width (cV)	83.94 (12.69–155.20)	0.023*	42.68 (−122.55–207.91)	0.60	2309.70 (−1537.06–6156.46)	0.23	−247.14 (−1305.99–811.72)	0.63
Abs. eosinophil (10∧9/L)	4.15 (0.79–7.51)	0.018*	−1.17 (−9.51–7.16)	0.77	134.32 (−45.59–314.24)	0.14	63.29 (18.69–107.89)	0.0080*
Abs. basophil (10∧9/L)	1.89 (0.23–3.54)	0.027*	0.97 (−2.45–4.40)	0.56	77.14 (−8.13–162.41)	0.074	5.87 (−16.07–27.82)	0.58
Bicarbonate level (mmol/L)	214.54 (41.93–387.15)	0.017*	−28.89 (−431.63–373.85)	0.88	8733.02 (−227.46–17693.51)	0.056	1700.47 (−753.55–4154.48)	0.16
Anion gap (mmol/L)	−183.24 (−289.11–−77.38)	0.0020*	0.27 (−288.19–288.73)	1.00	−8316.41 (−13802.0–−2830.85)*	0.0050*	−2294.72 (−3798.95–−790.50)	0.0050*
Sodium (mmol/L)	111.10 (−89.66–311.86)	0.26	54.92 (−389.96–499.80)	0.80	5829.30 (−4079.80–15738.40)	0.24	−346.36 (−3193.78–2501.05)	0.80

Linear regression models were used to estimate predictors of change in clinical features and were adjusted for age, sex, body mass index, and smoking pack-years. Inflammatory markers were used as independent variables to predict changes in all clinical features of patients. ß represents the change in a clinical feature associated with a one-unit change in biomarker expression.

*Denotes statistical significance.

MAP: mean arterial pressure.

## Discussion

Finding biomarkers of patient-relevant outcomes is a primary goal of COPD research [38]. Acute inflammatory changes that occur during exacerbations make it important to differentiate between biomarkers that might be useful for assessing disease activity and/or outcomes in stable-state from those found during exacerbation [38]. One such biomarker that offers potential in COPD is RelB, an NF-κB family member that is constitutively expressed in human lymphocytes and dendritic cells [39], suppresses cytokine production in lung epithelial cells [40] and is vital for thymus development and T cell function [41], [42]. Importantly, RelB suppresses CS-induced inflammation by interacting with the aryl hydrocarbon receptor (AhR) [23]–[27] to collectively control Cox-2 expression in lung fibroblasts [24]. Previous studies of the importance of RelB on CS and lung diseases have been restricted to experimental *in-vitro* and *in-vivo* models. Although these studies conclusively support an anti-inflammatory role for RelB against CS, there is no data on RelB expression in COPD or associations with relevant clinical outcomes. We report RelB expression for the first time in COPD patients and provide novel evidence that RelB may be associated with clinically-relevant features of COPD patients during exacerbations. Consequently, our study is an important step to provide insights into RelB expression and its potential role in COPD. It reports for the first time the associations between RelB and clinical parameters during COPD exacerbations.

One of our most intriguing findings is that although RelB mRNA expression was not associated with clinical outcomes in stable-state, at exacerbation RelB expression was negatively associated with several clinical parameters including systolic blood pressure. Our finding on RelB expression and systolic blood pressure is novel, as a relationship between RelB and blood pressure has not been reported to-date in humans. Experimentally RelB has been associated with balloon catheter injury in the rat carotid artery [43] and may be downregulated in response to treatment with DETA-NONOate- a nitric oxide donor [44]. Moreover, our recently published data show that RelB may suppress pulmonary ICAM-1 levels in response to CS [27]. When considered together with our current data, this supports a role for RelB in modulating endothelial function and blood pressure. This is potentially of importance, as it is well known that the period immediately following a COPD exacerbation is associated with increased risk of acute cardiovascular events [32]–[34]. In a separate analysis, we grouped stable-state and exacerbating subjects and found RelB expression to be negatively correlated to and able to predict negative changes in several outcomes including heart rate and PCO_2_, while predicting positive changes in pulse pressure (Table S1). Thus, identifying novel biological targets that could be capable of maintaining cardiovascular stability in COPD is of significant clinical value.

In addition to cardiovascular events, RelB expression was also related to anion gap, a parameter commonly used to identify acid-base disorders and disturbances [45]. In COPD, acid-base disturbances occur frequently [31] and lead to poor patient outcomes. Although it was recently shown that RelB is carbon dioxide-sensitive and contributes to the benefits of hypercapnia in pulmonary inflammatory diseases [30], our study is the first to report on RelB expression in relation to anion gap. This renders it possible that RelB may play a role in acid-base regulation during COPD exacerbations. Together, these data lend further support for an association between RelB and cardiovascular function as well as the involvement of RelB in acid-base maintenance. Thus, given the relationship between RelB and CS-induced inflammation, it might be reasonable to speculate that alterations in RelB expression and/or activity during exacerbations in COPD contribute to cardiovascular manifestations.

RelB dampens the expression of numerous COPD-relevant inflammatory mediators. Therefore we also examined associations between these (*i.e.* Cox-2, IL-8 and IL-1β) and patient clinical features. As with RelB expression, several of these mediators exhibited strong associations with cardiovascular alterations. Cox-2 is induced by HDL [46] and LDL [47], and although an association between Cox-2 and blood pressure has been described [48], [49], our study is first to suggest a relationship with cfPWV and AIx, two measures of arterial stiffening associated with cardiovascular risk. Moreover, the positive relationship between Cox-2 and RBC diameter width is novel and may be of clinical significance, as RBC diameter width-distribution is a powerful outcome predictor in chronic and/or acute left heart failure, and in COPD, can help identify right ventricle failure [50]. Associations between IL-8 and cardiovascular outcomes have been reported [51], [52], although none specifically on AIx and augmentation pressure. In our study IL-8 was also linked to acid-base parameters (pH, anion gap and PCO_2_) and fluctuations in these have been shown to alter pH and neutrophil IL-8 release [53]. Alterations in pH can also control IL-1β production by monocytes, not only supporting the relationship between IL-1β and pH [54] but also perpetuating and augmenting the inflammatory response in COPD.

Our study has strengths as well as limitations, the former including the fact that we recruited patients both in stable-state COPD and patients who were in acute exacerbation requiring hospitalization (*i.e.* severe exacerbation). Patients with severe COPD exacerbations are often difficult to recruit, as they are very sick and often refuse to participate in research studies. An additional strength of our study is that we also assessed patients within the first 72 hours of hospitalization, which allowed us to capture important physiological and clinical data that occur systemically and locally within the lung. A limitation of our study is that 25 of the 30 exacerbating patients took inhaled corticosteroids prior to blood collection. Corticosteroids can dampen Cox-2 and IL-8 expression via the NF-κB pathway by suppressing gene transcription [55], [56]. Thus the relative expression levels we report may be an under-representation. Another perceived limitation is the reliance on mRNA levels to correlate with clinical parameters, as quantification of blood RelB protein expression remains to be determined. However, RelB protein expression mirrors that of mRNA levels during dendritic cell differentiation [57]. Thus, we expect a similar association between blood RelB mRNA and protein levels in COPD. It would also strengthen our observations presented herein to further investigate the relationship between RelB expression and pulmonary patient outcomes in COPD stages GOLD I–IV; these investigations are currently ongoing. Finally, we recognize that the subjects in our study are unpaired, thereby reducing the statistical power and rendering it possible that inter-subject variability may have impacted our measurements. Thus, investigation of RelB and associated clinical outcomes in paired subjects (stable-state and exacerbation from the same subjects) is warranted. To ultimately determine the suitability of RelB as a biomarker in COPD, a longitudinal relationship between expression and associated outcomes must be examined.

There has been growing evidence that RelB is a potent suppressor of CS-induced inflammation [24], [25], [27], [40], [58]. Thus despite the limitations of our study, the expression and function of RelB in COPD represents a burgeoning area of research, and our data on the associations of RelB expression in COPD are highly novel and clinically relevant. Moreover, the results of our study suggest for the first time that blood RelB expression may be a noteworthy marker of cardiovascular events during COPD exacerbations. Future longitudinal and mechanistic studies will undoubtedly shed light on the functional significance of RelB in COPD pathogenesis and its potential for therapeutic modulation.

## Supporting Information

Figure S1 **Mean fold difference (± standard error) in RelB mRNA expression at exacerbation (n = 30) relative to RelB mRNA expression in stable-state patients (n = 18).** Fold decrease in RelB expression at exacerbation is 1.43 (p<0.001).
(DOCX)

Table S1 **Associations between RelB expression levels and clinical features of both exacerbating and stable-state patients (n = 48) and its ability to predict changes in these features.**
(DOCX)
