# Supplementary material for: Alterations in the Expression of the NF-κB Family Member RelB as a Novel Marker of Cardiovascular Outcomes during Acute Exacerbations of Chronic Obstructive Pulmonary Disease
Source: PLoS One. 2014 Nov 19;9(11):e112965. doi: 10.1371/journal.pone.0112965 (PMC4237338; doi:10.1371/journal.pone.0112965)
Supplement: Table S1 — Associations between RelB expression levels and clinical features of both exacerbating and stable-state patients (n = 48) and its ability to predict changes in these features. (DOCX) [file pone.0112965.s002.docx]

|  | | | | | | |
| --- | --- | --- | --- | --- | --- | --- |
|  | **Associations** | | | | **Ability to predict change in clinical features** | |
| **Clinical features** | **Correlation coefficient** | | **P-value** | | **ß (95% CI)** | **P-value** |
| Systolic BP | -0.42 | | 0.0030* | | -51205.3 (-89737.3 - -12673.4) | 0.010* |
| Diastolic BP | -0.33 | | 0.022* | | -30750.3 (-62720.6 - 1219.91) | 0.059 |
| Pulse (BPM) | -0.38 | | 0.0080* | | -43450.1 (-78570.1 - -8330.11) | 0.017* |
| O2 saturation (%) | 0.070 | | 0.62 | | 2282.89 (-4423.89 - 8989.67) | 0.50 |
| pH | -0.18 | | 0.33 | | -23.34 (-121.59 - 74.91) | 0.63 |
| PO2 (mmHg) | -0.12 | | 0.50 | | -3703.96 (-31894.9 - 24486.97) | 0.79 |
| PCO2 (mmHg) | 0.34 | | 0.050* | | 11295.79 (-3690.84 - 26282.43) | 0.13 |
| Na+ (mmol/L) | 0.29 | | 0.11 | | 6364.29 (-5672.46 - 18401.04) | 0.29 |
| K+ (mmol/L) | -0.29 | | 0.11 | | -2956.32 (-6340.64 - 428.00) | 0.084 |
| Ca++ (mmol/L) | 0.15 | | 0.42 | | 22.72 (-115.24 - 160.68) | 0.74 |
| Hct (%) | -0.14 | | 0.44 | | -10363.1 (-26522.9 - 5796.77) | 0.20 |
| Approximate number of exacerbations reported during the previous year | -0.1 | | 0.51 | | -1009.36 (-4995.93 - 2977.21) | 0.62 |
| FEV1 L | 0.11 | | 0.46 | | -54.56 (-863.62 - 754.49) | 0.89 |
| FEV1 % predicted | -0.07 | | 0.63 | | -18874.4 (-49897.7 - 12148.90) | 0.23 |
| FEV1/FVC | -0.22 | | 0.15 | | -300.82 (-569.17 - -32.48) | 0.029* |
| mean cfPWV (m/s) | 0.15 | | 0.34 | | 2012.17 (-9002.75 - 13027.09) | 0.71 |
| mean MAP (mmHg) | -0.2 | | 0.21 | | -16046.9 (-50007.2 - 17913.50) | 0.34 |
| mean PP (mmHg) | 0.31 | | 0.051 | | 29042.30 (3063.65 - 55020.94) | 0.030* |
| mean AP (mmHg) | 0.24 | | 0.14 | | 13136.28 (-971.24 - 27243.80) | 0.067 |
| mean Aix (%) | 0.15 | | 0.34 | | 18482.26 (-10646.3 - 47610.86) | 0.21 |
| mean crPWV 1 (m/s) | 0.12 | | 0.44 | | 841.56 (-2867.24 - 4550.37) | 0.65 |
| Cholesterol (mmol/L) | -0.15 | | 0.32 | | -511.91 (-3010.68 - 1986.86) | 0.68 |
| Triglycerides (mmol/L) | 0.040 | | 0.82 | | 272.50 (-1368.49 - 1913.49) | 0.74 |
| HDL (mmol/L) | -0.14 | | 0.38 | | -156.49 (-1425.70 - 1112.71) | 0.80 |
| LDL (mmol/L) | -0.12 | | 0.46 | | -478.54 (-2746.65 - 1789.57) | 0.67 |
| Cholesterol/HDL | 0.030 | | 0.82 | | -54.97 (-2426.23 - 2316.29) | 0.96 |
| WBC (10^9/L) | -0.12 | | 0.42 | | -2813.16 (-12962.0 - 7335.69) | 0.58 |
| RBC (10^12/L) | -0.06 | | 0.70 | | -737.39 (-2077.71 - 602.93) | 0.27 |
| Hemoglobin (g/L) | -0.18 | | 0.23 | | -31628.3 (-71677.7 - 8421.00) | 0.12 |
| Hct (L/L) | -0.14 | | 0.35 | | -80.22 (-198.90 - 38.46) | 0.18 |
| Mean cell vol. (fL) | -0.14 | | 0.36 | | -3049.93 (-17685.5 - 11585.62) | 0.68 |
| Mean cell hemoglobin (pg/cell) | -0.18 | | 0.23 | | -2086.62 (-7842.80 - 3669.56) | 0.47 |
| Mean cell hemoglobin Conc. (g/L) | -0.2 | | 0.17 | | -11957.5 (-33895.0 - 9980.04) | 0.28 |
| RBC diameter Width (cV) | 0.29 | | 0.054 | | 2650.85 (-1663.19 - 6964.90) | 0.22 |
| Platelet (10^9/L) | -0.18 | | 0.24 | | -73614.0 (-271159 - 123931.5) | 0.46 |
| Platelet Hct | -0.13 | | 0.40 | | -37.11 (-189.86 - 115.64) | 0.63 |
| Mean platelet vol. (fL) | 0.16 | | 0.30 | | 563.72 (-1165.91 - 2293.35) | 0.51 |
| Platelet Dist. Width (cV) | 0.030 | | 0.86 | | 342.31 (-838.73 - 1523.34) | 0.56 |
| Abs. Lymphocyte (10^9/L) | 0.33 | | 0.025* | | 1463.58 (-10.50 - 2937.66) | 0.052 |
| Abs. MNC (10^9/L) | 0.20 | | 0.18 | | 239.08 (-572.63 - 1050.80) | 0.56 |
| Abs. Neutrophil (10^9/L) | -0.2 | | 0.17 | | -5032.65 (-15249.6 - 5184.34) | 0.33 |
| Abs. Eosinophil (10^9/L) | 0.44 | | 0.0020* | | 273.19 (39.32 - 507.06) | 0.023* |
| Abs. Basophil (10^9/L) | 0.24 | | 0.11 | | 80.54 (-8.99 - 170.08) | 0.077 |
| Bicarbonate Level (mmol/L) | 0.36 | | 0.016* | | 7183.79 (344.14 - 14023.45) | 0.040* |
| Anion Gap (mmol/L) | -0.46 | | 0.0010* | | -7400.53 (-12612.0 - -2189.07) | 0.0070* |
| Creatinine (umol/L) | -0.08 | | 0.62 | | -21089.4 (-76711.5 - 34532.62) | 0.45 |
| Glucose Random (mmol/L) | -0.45 | | 0.0020* | | -11766.3 (-20056.2 - -3476.45) | 0.0070* |
| Chloride (mmol/L) | 0.20 | | 0.20 | | 6280.30 (-3149.26 - 15709.86) | 0.19 |
| Potassium (mmol/L) | -0.09 | | 0.58 | | -131.31 (-1059.12 - 796.51) | 0.78 |
| Sodium (mmol/L) | 0.27 | | 0.069 | | 6063.56 (-1496.57 - 13623.70) | 0.11 |
| Pearson correlation coefficients were used to determine associations. | | |  | |  |  |
| Linear regression models were used to estimate predictors of change in clinical features and were adjusted for age, sex, body mass index, and smoking pack-years. RelB was used as an independent variable to predict changes in all clinical features of patients. ß represents the change in a clinical feature associated with a one-unit change in RelB expression. | | | | | | |
| *Denotes statistical significance p<0.05. | |  | |  |  |  |
| BPM: beats per minute, O2: oxygen, PO2: partial pressure of oxygen, PCO2: partial pressure of carbon dioxide, Na+: sodium, K+: potassium, Ca++: calcium, Hct: hematocrit, FEV1: forced expiratory volume in 1 second, FVC: forced vital capacity, BP: blood pressure, cfPWV: carotid-femoral pulse wave velocity, MAP: mean arterial pressure, PP: pulse pressure, AP: augmentation pressure, Aix: augmentation index, crPWV: carotid-radial pulse wave velocity, HDL: high-density lipoprotein, LDL: low-density lipoprotein, WBC: white blood cell, RBC: red blood cell, vol.: volume, Abs.: absolute, MNC: monocyte. | | | | | | |
