# Supplementary material for: Alterations in the Expression of the NF-κB Family Member RelB as a Novel Marker of Cardiovascular Outcomes during Acute Exacerbations of Chronic Obstructive Pulmonary Disease
Source: PLoS One. 2014 Nov 19;9(11):e112965. doi: 10.1371/journal.pone.0112965 (PMC4237338; doi:10.1371/journal.pone.0112965)
Supplement: Supplementary file 2 [file pone.0112965.s001.docx]

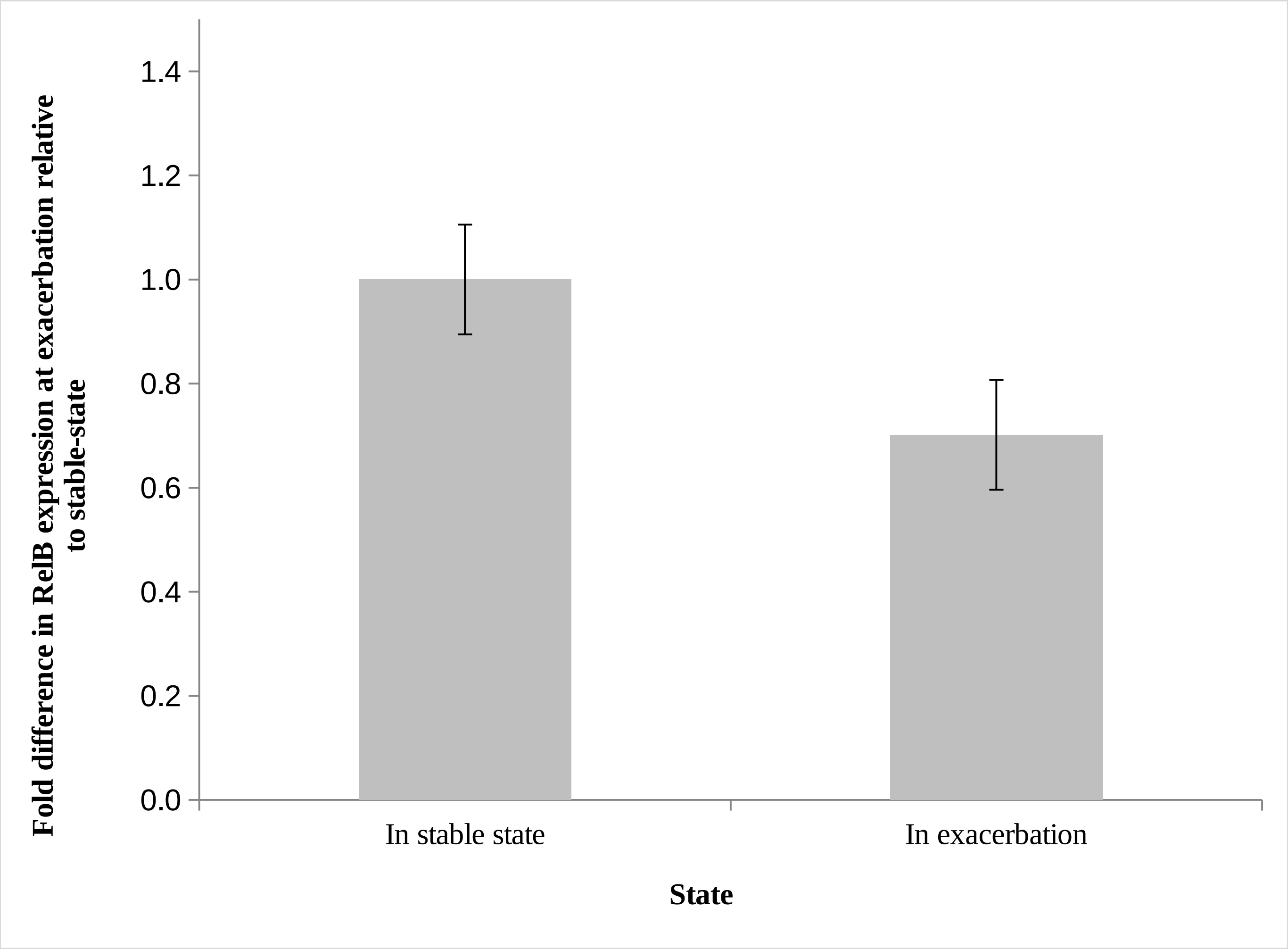
Fold difference in RelB expression at exacerbation relative to stable-state
1.4
1.2
1.0
0.8
0.6
0.4
0.2
0.0
In stable state
In exacerbation
State
